# The Archean atmosphere

**DOI:** 10.1126/sciadv.aax1420

**Published:** 2020-02-26

**Authors:** David C. Catling, Kevin J. Zahnle

**Affiliations:** 1 Department of Earth and Space Sciences and cross-campus Astrobiology Program, Box 351310, University of Washington, Seattle, WA 98195, USA.; 2 Space Sciences Division, NASA Ames Research Center, MS 245-3, Moffett Field, CA 94035, USA.

## Abstract

The atmosphere of the Archean eon—one-third of Earth’s history—is important for understanding the evolution of our planet and Earth-like exoplanets. New geological proxies combined with models constrain atmospheric composition. They imply surface O_2_ levels <10^−6^ times present, N_2_ levels that were similar to today or possibly a few times lower, and CO_2_ and CH_4_ levels ranging ~10 to 2500 and 10^2^ to 10^4^ times modern amounts, respectively. The greenhouse gas concentrations were sufficient to offset a fainter Sun. Climate moderation by the carbon cycle suggests average surface temperatures between 0° and 40°C, consistent with occasional glaciations. Isotopic mass fractionation of atmospheric xenon through the Archean until atmospheric oxygenation is best explained by drag of xenon ions by hydrogen escaping rapidly into space. These data imply that substantial loss of hydrogen oxidized the Earth. Despite these advances, detailed understanding of the coevolving solid Earth, biosphere, and atmosphere remains elusive, however.

## Teaser

What was the early atmosphere made of? We review what is known during the Archean eon, 4 to 2.5 billion years ago.

## INTRODUCTION: BACKGROUND TO ARCHEAN EARTH

**Fig. 1:**
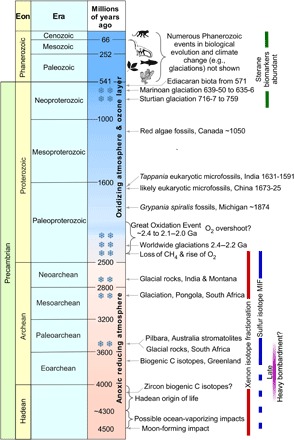
Precambrian events and atmospheric change. For biological evolutionary dates, see (*6*). For a description of other events, see (*64*) and references therein.

The environment of the Archean eon from 4 to 2.5 billion years (Ga) ago has to be understood to appreciate biological, geological, and atmospheric evolution on our planet and Earth-like exoplanets (Fig. 1) [e.g., (*1*, *2*)]. Its most distinguishing characteristic was negligible O_2_, unlike today’s air, which contains, by dry volume, 21% O_2_, 78% N_2_, 0.9% Ar, and 0.1% other gases. With its radically different atmosphere and lack of macroscopic, multicellular life, the Archean world was alien. However, at that time, the beginnings of modern Earth emerged. For example, cyanobacteria probably evolved [e.g., (*3*)] during this period, and these oxygenic photoautotrophs eventually oxygenated the air, setting the stage for later, complex life, including us (*4*).

The earliest well-preserved sedimentary and volcanic rocks are Archean and provide insights into atmospheric composition, climate, and life. These perspectives are unavailable for the Hadean eon from ~4.6 to 4 Ga ago, which generally lacks these rocks. For context, the Archean precedes the Proterozoic eon of 2.5 Ga to 541 ± 1 million years (Ma) ago, and Archean eras provide a timeline for our discussion: the Eoarchean (4 to 3.6 Ga ago), Paleoarchean (3.6 to 3.2 Ga ago), Mesoarchean (3.2 to 2.8 Ga ago), and Neoarchean (2.8 to 2.5 Ga ago).

The Archean was originally conceived to span the time from after the origin of life to the advent of free O_2_ (*5*). While the origin of life dates back to before 3.5 to 3.8 Ga ago or earlier [e.g., (*6*)], newer information puts atmospheric oxygenation after ~2.4 Ga ago, inside the Proterozoic. Here, considering the Archean in the older sense, the origin of life falls outside this Review, while the story of oxygen’s rise falls within.

Data about the Archean atmosphere come from how individual gases, or the air as a whole, affected chemical and physical phenomena (e.g., the composition of aerosols, chemical reactions in soils, raindrop terminal velocity, isotopic fractionations, etc.) that were recorded in rocks. So, after a brief discussion of the Hadean, we review what the Archean atmosphere was made of. However, because of limited proxy data, major uncertainties remain about the exact levels of atmospheric gases over time.

Our discussion also considers evidence for early life and its possible global influence. We assume that metabolically useful gases would have been consumed, while waste gases would have been excreted, as they are today.

Oxygenic photosynthesis produced the most impactful waste gas. The O_2_ from cyanobacterial ancestors flooded the atmosphere rapidly at a time between 2.4 and 2.3 Ga, with the transition marked in the rocks by the sudden disappearance of mass-independent fractionation (MIF) of sulfur isotopes (discussed in detail later) (*7*, *8*). The Great Oxidation Event (GOE) thus began, which ended ~2.1 to 2.0 Ga ago (*9*, *10*). Although this switch to an oxygenated atmosphere and shallow ocean occurred in the Paleoproterozoic era (2.5 to 1.6 Ga ago), the weakly reducing atmosphere that was eliminated typified the Archean [e.g., (*11*)]. Here, “weakly reducing” means minor levels of reducing gases, such as CO, H_2_, and CH_4_, in an anoxic atmosphere of bulk oxidized gases, CO_2_ and N_2_. A major outstanding question concerns how trends of biological and geological evolution relate to the GOE.

Atmospheric composition, in turn, affected Archean climate. At 4 Ga ago, solar luminosity was 25 to 30% lower than today (*12*, *13*), but Archean Earth was not persistently frozen because abundant evidence shows an active hydrological cycle. Liquid water under a fainter Sun likely implies more abundant greenhouse gases than today (*14*, *15*). We review what the gases were and their levels.

In addition to a gradual increase in solar luminosity, slow changes in the solid Earth over time provided boundary conditions for atmospheric evolution. On geological time scales, volcanic and metamorphic gases replenish atmospheric volatiles that escape to space or are chemically sequestered into solid materials.

We will not attempt to resolve controversies over how much solid Earth evolution drove atmospheric evolution. Consequently, we omit the large topic of how Earth’s outgassing history depended on debated tectonic and geological evolution models.

## A BRIEF OVERVIEW OF THE ENVIRONMENT BEFORE THE ARCHEAN

The composition of the Hadean atmosphere is obscured by a lack of well-preserved rocks, but analysis of zircons—crystals of zirconium silicate (ZrSiO_4_)—suggests that continents, oceans, and perhaps life all originated in the Hadean (*16*). Zircons are tiny (<0.5 mm) durable pieces of continental crust. Elevated ^18^O/^16^O in 4.3-Ga-old zircons was possibly inherited from ^18^O-enriched, weathered surface rocks that were later buried and melted, which implies the presence of surficial liquid water and even land (*17*, *18*). In addition, graphite inside a 4.1-Ga-old zircon has a biogenic-like δ^13^C of −24 per mil (‰) (*19*), although lack of context means that an abiotic origin cannot be eliminated. [Here, δ^13^C is the ^13^C/^12^C ratio of a sample in parts per thousand (‰) relative to a standard reference material: δ^13^C = 1000 × [(^13^C/^12^C)_sample_/(^13^C/^12^C)_standard_ − 1]].

Impact bombardment would have affected Hadean and subsequent Archean environments. The lunar record implies a decay of terrestrial impact bombardment extending into the Archean (*20*, *21*). The estimated median age of the last impact big enough to vaporize the entire ocean is ~4.3 Ga ago (*22*), which provides a crude upper age limit on the origin of life. An origin of life during the period ~4.3 to 4.0 Ga ago is consistent with phylogenetic inferences [e.g., (*23*)]. Later, the Late Heavy Bombardment (LHB) is a hypothesized interval of enhanced bombardment superposed on the general decline, which occurred between 4.2 and 4.0 to ~3.5 Ga ago, based on the ages of lunar rocks and meteorite shocks (*24*), although many dispute that the LHB was a discrete event (*25*). Regardless, an LHB would likely not sterilize Earth (*26*); any microbial life would have rebounded (*27*).

The mantle contains excess highly siderophile elements (HSEs) relative to concentrations expected after Earth’s iron core formed, which removed HSEs. Similarities of isotopes and relative proportions of these HSEs to those in enstatite chondrite and achondrite meteorites suggest that this highly reducing meteoritic material was delivered late in Earth’s accretion (*28*). Thus, carbon and nitrogen were supplied in graphite and nitrides. Therefore, the Hadean atmosphere and mantle were probably initially highly reducing, before subsequent oxidation either by hydrogen escape (*29*) or disproportionation of mantle FeO accompanied by Fe loss to the core (*30*, *31*). In any case, iron-cored impactors would reduce seawater to hydrogen and create transient, highly reducing atmospheres that may have been important for the origin of life (*32*, *33*).

Because a liquid ocean likely existed by ~4.4 Ga ago, feedbacks in the geologic carbon cycle (discussed later) probably stabilized the long-term climate (*34*). However, consumption of CO_2_ in the weathering of impact ejecta by carbonic acid suggests a cool early Hadean surface near 0°C under the faint Sun (*35*, *36*).

Estimates of nitrogen bound in today’s solid Earth range a few to ~40 bar equivalent (*37*, *38*), which allows for considerable N_2_ in the Hadean atmosphere unless N was incorporated into a reducing, deep layer of magma—a magma ocean—formed after the Moon-forming impact ~4.5-Ga ago [(*39*) and references therein]. So, although N_2_ was one of the bulk atmospheric gases, its Hadean level—higher than today or lower—remains unclear.

In summary, at the end of the Hadean, Earth had oceans, continents (*16*), an anoxic atmosphere likely rich in CO_2_ and N_2_, and probably life (Fig. 1).

## WHAT WAS THE ARCHEAN ATMOSPHERE MADE OF?

Proxies constrain Archean atmospheric composition. Gases reacted with the seafloor or land, leaving chemical traces in seafloor minerals (*40*, *41*) or in soils that became paleosols (*42*, *43*). In addition, atmospheric particles carried isotopic signatures into sediments that were diagnostic of atmospheric composition (*44*–*47*). Occasionally, fluid inclusions in rocks trapped seawater with dissolved air (*48*–*50*) or even microbial gases (*51*).

Sometimes the physical environment affected rocks and minerals. Their preservation allows estimates of environmental temperature (*52*–*55*) and barometric pressure (*56*, *57*).

Table 1 summarizes inferences about the composition of the Archean atmosphere and ocean. Here, gas concentrations are generally for the base of the troposphere. Although the Archean lacked a stratospheric ozone layer, it remains valid to refer to a troposphere and stratosphere as vertical regions where, respectively, convection and radiation dominated the energy transfer.

**Table 1:** Archean environmental constraints. Constraints on atmospheric gases at ground level (unless stated otherwise) and some bulk marine species. Gas level constraints are given in the same units as in the cited papers: partial pressure in bar or atm, where 1 bar = 0.9869 atm, or as mixing ratios (ppmv = parts per million by volume; S-MIF = sulfur isotope mass-independent fractionation).

**Archean atmospheric gases**			
**Parameter or species**	**Published constraint**	**Age (years before present)**	**Basis of constraint**
O_2_	<10^−6^ × present O_2_	>2.4 Ga	Modeled S_8_ flux needed to create and carry S-MIF (*44*)
	<3.2 × 10^−5^ atm	2.415 Ga	Detrital uraninite (*66*), if the river length feeding the Koegas subgroup is accurately estimated
O_3_ column	<10^15^ molecules m^−2^	>2.4 Ga	Modeled for ground-level O_2_ < 0.2 ppmv (*44*, *270*)
Surface barometric pressure	<0.52–1.1 bar	2.7 Ga	Maximum fossil raindrop imprint size (*57*)
	0.23 ± 0.23 bar (2σ)	2.74 Ga	Fossil vesicles at the top and base of basaltic lavas (*56*)
N_2_	<1.1 bar (2σ)	3.5–3.0 Ga	N_2_/^36^Ar in fluid inclusions (*48*)
	<1 bar (2σ)	3.3 Ga	Derived from Fig. 4A and Table 2 of Avice *et al.* (*49*).
HCN	Up to ~100 ppmv	>2.4 Ga	Photochemistry if CH_4_ was ~10^3^ ppmv (*166*, *167*)
N_2_O	~Few ppbv	>2.4 Ga	Lightning production of NO and HNO in a reducing atmosphere, dissolution of HNO, and evaporation
CO_2_	>0.0004 bar (0°C), >0.0025 bar (25°C), >0.26 bar (100°C)	3.2 Ga	Siderite weathering rinds on river gravel (*257*)
	0.03–0.15 bar	2.77 Ga	Mt. Roe paleosol, Australia (*43*)
	0.02–0.75 bar	2.75 Ga	Bird paleosol, South Africa (*43*)
	0.003–0.015 bar	2.69 Ga	Alpine Lake paleosol, MN, United States (*168*)
	0.05–0.15 bar	2.46 Ga	Pronto/NAN paleosol, Canada (*43*)
	<~0.8 bar	3.8–2.4 Ga	Enough UV to make S-MIF (*58*)
CH_4_	>20 ppmv	>2.4 Ga	Lower limit for sufficient reductant for S-MIF (*44*)
	>~5000 ppmv	~3.5 Ga	Enough methane to induce sufficiently rapid hydrogen escape to drag Xe^+^ and fractionate Xe isotopes (*187*)
CO	Less than a few ppmv	After the origin of CO consumers	Thermodynamic limit if microbes used available free energy of CO [appendix A of Krissansen-Totton *et al.* (*250*)]
	<300 ppmv		Limit if transfer of gas through the atmosphere-ocean interface restricts CO consumption (*183*).
H_2_	10 s–100 ppmv	After the origin of methanogens	Assuming that methanogens used available free energy from consuming H_2_ (*197*, *271*) and the transfer of gas through the atmosphere-ocean interface restricts H_2_ consumption (*183*).
	<0.01 bar	3.0–2.7 Ga	The survival of detrital magnetite in rivers, if Fe^3+^-reducing microbes are assumed (*200*)
**Archean seawater species**			
pH	6.4–7.4 6.75–7.8	At 4 Ga At 2.5 Ga	95% confidence ranges from a carbon cycle model with 10^4^-ppmv Archean CH_4_ (*34*).
SO_4_^2−^(aq) (bulk sea)	<2.5 μM	>2.4 Ga	Lack of mass-dependent sulfur isotope fractionation (*75*)
NO_3_^−^(aq) (bulk sea)	~0	>2.4 Ga	By analogy to the deep, anoxic Black Sea (*272*)
NH_4_^+^(aq)	0.03–10.3 mM (probably porewater rather than seawater)	~3.8 Ga	From the N content of biotites (originally clays), derived from adsorption of dissolved NH_4_^+^(aq) (*148*)
Fe^2+^(aq) (deep sea)	40–120 μM (2–7 ppm by weight)	>2.4 Ga	Based on solubility constraints of Fe^2+^ (*139*)
Salinity (g/kg)	~20–50 at 40°–0°C versus modern of 35	3.5–3.0 Ga	From seawater fluid inclusions in quartz (*50*)
Potassium	Cl/K ~50 versus modern 29	3.5–3.0 Ga	From seawater fluid inclusions in quartz (*50*)

### Negligible Archean O_2_ but oxygen oases after oxygenic photosynthesis evolved

**Fig. 2:**
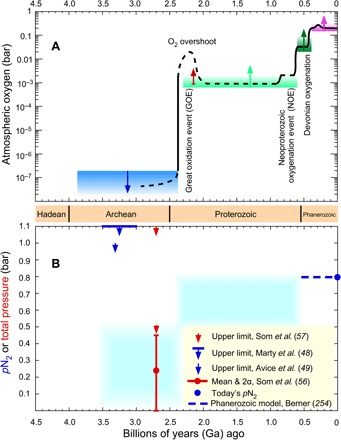
Schematic histories of atmospheric O_2_ and surface barometric pressure or N_2_. (**A**) Colored arrows faithfully represent known O_2_ constraints, but the black line is speculative. An Archean upper bound of <0.2-μbar O_2_ (blue) is for photochemistry that generates S_8_ aerosols, preserving observed mass-independent isotope fractionation in sulfur compounds (*44*). The size and shape of an O_2_ overshoot during the GOE are highly uncertain; a lower bound (red arrow) comes from iodine incorporation into carbonates (*251*). In the Proterozoic, a lower bound (light green) of 6 × 10^−4^ bar is required for an O_2_-rich atmosphere to be photochemically stable (*44*). However, O_2_ levels likely remained low for most of the Proterozoic (*252*). Neoproterozoic oxygenation began around ~800 Ma ago. From ~600 Ma ago, a lower bound of >0.02-bar O_2_ (dark green) is from plausible O_2_ demands of macroscopic Ediacaran and Cambrian biota (*120*). Charcoal since 0.4 Ga ago implies a lower bound of >0.15 bar (purple) (*253*). The post-Devonian black line for O_2_ evolution approximately represents curves from calculations of C and S isotopic mass balance (*254*, *255*). (**B**) Constraints on surface atmospheric pressure (red) (*56*, *57*) and the partial pressure of nitrogen, *p*N_2_ (blue) (*48*, *49*, *140*). Blue shading shows a schematic and speculative *p*N_2_ range in different time intervals consistent with very sparse proxy data.

The strongest constraint on Archean atmospheric composition is that the ground-level mixing ratio of O_2_ was <10^−6^ PAL (present atmospheric level) or <0.2–parts per million by volume (ppmv) O_2_ for air of 1 bar, indicated by the presence of sulfur isotope MIF (S-MIF) in Archean sedimentary minerals (Fig. 2A) (*44*, *47*). An oft-quoted limit of 10^−5^ PAL O_2_ derives from an earlier photochemical model that could not address O_2_ levels in the range of 10^−5^ to 10^−15^ PAL (*45*). Usually, isotope fractionation is proportional to the mass difference between isotopes; e.g., in diffusive separation of sulfur-containing gases, ^34^S becomes about half as abundant, relative to ^32^S, as ^33^S. However, some particular photochemical reactions produce MIF that, by definition, deviates from proportionality to mass.

Archean S-MIF is tied to the production of elemental sulfur, S_8_, from photochemistry in anoxic air; in contrast, in an oxic atmosphere, S-MIF nearly disappears. Photochemistry imparts S-MIF and produces elemental sulfur, starting with reactions that photolyze volcanic SO_2_ such as SO_2_ + *h*ν (<217 nm) = SO + O and SO + *h*ν (< 231 nm) = S + O (*58*, *59*). This photolysis occurs when short-wavelength ultraviolet (UV) penetrates Earth’s troposphere in the absence of a stratospheric ozone (O_3_) layer. Scarce ozone implies negligible O_2_ from which O_3_ derives.

In anoxic air, sulfur ends up in insoluble S_8_ aerosols and water-soluble sulfate and SO_2_, unlike today’s atmospheric sulfur, which almost entirely oxidizes to sulfate (*44*, *45*). In the Archean, as well as anoxia, gases such as CH_4_ or H_2_ produced sufficiently reducing conditions that S, S_2_, S_3_, etc. gases persisted and polymerized into S_8_ (*44*, *60*). Reactions that dominantly imparted S-MIF are debated: They include polysulfur formation (*61*), SO_2_ photolysis, and other reactions (*46*, *59*). In any case, when S_8_ particles fell to Earth’s surface, their S-MIF isotopic composition complemented that of sulfate particles, allowing preservation of different S-MIF signs in these phases as sedimentary pyrite (FeS_2_) and barite (BaSO_4_) (*62*). Sulfate, of course, could later be microbially transformed to pyrite.

Numerous redox-sensitive tracers corroborate negligible Archean O_2_ [reviewed, e.g., in (*10*, *63*, *64*)]. Anoxia proxies that have long been recognized include the lack of pre-GOE continental sediments stained by red ferric oxides (redbeds), detrital grains from well-aerated rivers of siderite (FeCO_3_), uraninite (UO_2_), or pyrite that would oxidize and dissolve or rust at high pO_2_ (*65*, *66*), and paleosols with iron washed out by anoxic rainwater [e.g., (*67*)]. Furthermore, Archean marine sediments have low concentrations of elements that enter rivers during oxidative continental weathering (*68*–*70*). Conversely, glacial sediments contain continental materials lacking oxidative weathering loss of molybdenum (*71*).

Iron formations (IFs), which are marine chemical sedimentary rocks rich in iron and silica [15 to 40 weight % (wt %) Fe and 40 to 60 wt % SiO_2_], indicate that the deep Archean ocean contained Fe^2+^(aq) and so was anoxic [e.g., (*72*)]. In “Superior-type IFs” that formed near-shore, rare earth elements show that dissolved iron was partly sourced from seafloor hydrothermal vents and upwelled onto continental shelves where the iron precipitated. In today’s deeply oxygenated oceans, oxidized iron instead precipitates locally around vents. Archean shallow-water IFs constrain atmospheric O_2_ to <2.4 × 10^−4^ bar (*73*).

The absence or presence of mass-dependent fractionation of various isotopes can also indicate anoxic versus oxic conditions. In the case of sulfur, today’s oxidative weathering of continental sulfides produces soluble sulfate, which rivers carry to the ocean. When bacteria reduce sulfate to pyrite in seafloor sediments, they impart mass-dependent S isotope fractionation if sulfate is present at sufficient concentrations as in the modern oceans, but usually not in Archean seawater (*74*, *75*). The absence of this isotope fractionation indicates little Archean seawater sulfate and implies anoxic air. Using similar arguments of O_2_-sensitive weathering, transport, and fractionation, isotopes of Cu (*76*), Cr (*77*), Fe (*78*), U (*79*), Mo [e.g., (*80*, *81*)], and Se (*82*) indicates an anoxic Archean atmosphere.

Atmospheric oxidation of 2.7-Ga-old iron-nickel micrometeorites has been used to argue for O_2_ near-modern levels above ~75-km altitude (*83*, *84*). However, given copious evidence for an anoxic Archean atmosphere, an alternative explanation is that high CO_2_ levels (perhaps >70%) oxidized the micrometeorites (*85*).

Nonetheless, even under a globally anoxic atmosphere, lakes and shallow seawater inhabited by oxygenic photosynthesizers could have become “oxygen oases”—local or regional areas with elevated O_2_. Modern surface seawater dissolves 0.25 mM O_2_ at 15°C, while estimates for Archean oxygen oases range from 0.001 to 0.017 mM (0.4 to 7% of present) (*86*, *87*).

When exactly oxygenic photosynthesis began and dominated over anoxygenic photosynthesis is debated, but signs of biological carbon fixation appear early. Graphite in a ~3.7-Ga-old outcrop of sedimentary rock is ^12^C-enriched in amounts typical of photosynthetic microbes (*88*, *89*). Then, at 3.52 Ga ago, δ^13^C in kerogen of −24‰ and associated marine carbonate of −2‰ found in Australia is similar to biological isotope fractionation in modern oceans (*90*).

Fossil evidence for Archean cyanobacteria is reported. Light organic carbon isotopes and structures like those made by filamentous cyanobacteria found within stromatolites or other microbially induced sedimentary structures are consistent with cyanobacteria by 3.2 to 2.7 Ga ago (*91*–*94*). Cyanobacteria could be corroborated by biomarkers, which are remnant organic molecules from particular organisms. However, putative Archean biomarkers have been plagued by younger contamination [e.g., (*95*)].

Instead, cyanobacterial interpretations are strengthened by geochemical data suggesting O_2_ oases at 3.2 to 3.0 Ga ago. Fractionated iron and molybdenum isotopes and levels of redox-sensitive metals suggest marine photic zone O_2_ (*96*–*99*). [Chromium isotope data have been used to argue for the existence of ~3 Ga-old terrestrial oxygen (*100*), but they were probably caused by modern oxidative weathering (*101*).]

Then, by 2.8 to 2.6 Ga ago, increasing concentrations and isotopic fractionation of Mo and S in marine shales suggest that O_2_ proximal to cyanobacterial mats and stromatolites on land oxidized sulfides and boosted sulfate and Mo riverine fluxes to the oceans (*70*, *80*, *102*–*104*). These trends are consistent with isotopic evidence for Neoarchean methanotrophy and oxidative nitrogen cycling (*105*–*109*).

Concentration spikes at 2.5 to 2.66 Ga ago in Mo, Se, and Re and isotopic excursions of Mo, Se, U, and N have been interpreted as arising from O_2_ transients or “whiffs” of O_2_ (*110*–*113*). Critics argue that the data derive from post-GOE alteration (*114*, *115*) [but see (*116*)]. Alternatively, oxygen oases of cyanobacteria within soils or lakebeds may have mobilized these elements into rivers and then the sea (*117*).

Phylogenetic analyses mostly suggest that cyanobacteria originated by 2.8 Ga. Molecular clocks must be calibrated by physical evidence, and phylogenetic methods are themselves debated. While some argue that oxygenic photosynthesis evolved only 50 to 100 Ma before the GOE (*115*), most studies suggest an earlier Paleoarchean or Mesoarchean age [e.g., (*3*, *118*, *119*)].

A major unresolved issue is how the GOE was related to underlying geological or biological trends (Fig. 2A). Life on its own cannot change the net redox state of the global environment because each biological oxidant is complemented by a reductant. In oxygenic photosynthesis, a mole of organic carbon accompanies every mole of O_2_

$${\text{CO}}_{2}+\;H_{2}O\overset{\text{photosynthesis}}{\underset{\text{respiration},\text{decay}}{⇄}}{\text{CH}}_{2}O+O_{2}$$ (1)

Today, with ~4.4 × 10^5^–Tmol surface organic matter and ~9000 Tmol/year of oxidative decay or respiration (*120*), reaction 1 reverses in ~50 years. Consequently, for long-term O_2_ accumulation, some organic carbon must be segregated from the O_2_ and buried. Alternatively, O_2_ molecules are liberated if microbes use the organic carbon from reaction 1 to make other reductants, such as sulfides from sulfates, that are buried. However, Archean seawater sulfate concentrations were small (Table 1), so organic carbon burial is the O_2_ flux that matters for the GOE. Atmospheric oxidation also occurs when hydrogen escapes to space after the photochemical breakdown of gases such as H_2_ and CH_4_, which ultimately derive from water and are relatively abundant in anoxic air.

Atmospheric O_2_ is determined by net redox fluxes into and out of the atmosphere. This simple truth is deceptive because these redox fluxes are themselves controlled by less easily constrained oxidative weathering (both seafloor and continental) and volcanic and metamorphic degassing, as well as hydrogen escape to space. Permanent atmospheric oxygenation requires ~3 × 10^−3^ PAL O_2_ or more to prevent a destabilizing positive feedback of photochemical destruction of tropospheric O_2_ that otherwise occurs when an incipient ozone column is still transparent to far UV (*44*, *121*, *122*).

These O_2_ levels would have only been attained when the O_2_ flux from the burial of organic carbon exceeded the kinetically efficient sink from O_2_-consuming gases (CO, H_2_, H_2_S, and SO_2_) from volcanism and metamorphism plus fluxes of reducing cations such as Fe^2+^ from seafloor vents (*9*, *120*, *121*, *123*, *124*). A minority assume that such a flux imbalance applied as soon as oxygenic photosynthesis evolved, mandating a rapid rise of O_2_ (*125*). In the consensus assessment that oxygenic photosynthesis evolved long before the GOE, efficient consumption of O_2_ initially suppressed O_2_ levels.

Hypotheses about the GOE tipping point are reviewed elsewhere [(*64*), chap. 10]. Briefly, ideas favoring increased O_2_ fluxes from organic burial appeal to more continental shelf area available for burial (*126*), more phosphorus to stimulate photosynthesis (*127*, *128*), or subduction of organic carbon relative to ferric iron (*129*). Because organic carbon burial extracts ^12^C and leaves inorganic carbonates ^12^C depleted, it is difficult to reconcile these hypotheses with the remarkable constancy of the carbon isotope record, which indicates little change in average organic burial rates between the Archean and Proterozoic (*130*). However, an increase in organic burial might have occurred if negligible oxidative weathering of ^12^C-rich organics on land or a sink of seafloor carbonate meant that the operation of the carbon cycle or the isotopic composition of carbon input into the surface environment differed from today (*130*–*132*).

Hypotheses for a slowly decreasing O_2_ sink to the GOE tipping point rely on a decline in the ratio of reduced-to-oxidized species from volcanic, metamorphic, and hydrothermal sources (*9*, *123*, *124*, *133*, *134*). Some emphasize the role of hydrogen escape to space in oxidizing solid Earth, lowering Earth’s capacity to release O_2_-consuming reductants (*121*, *135*, *136*). New evidence from xenon isotopes supports rapid Archean hydrogen escape, as discussed below.

### Nitrogen gases in the Archean

Molecular nitrogen dominates today’s atmosphere, and three lines of evidence have begun to constrain Archean N_2_ levels (Fig. 2B). First, the largest size of 2.7-Ga-old fossil raindrop imprints provides a conservative limit of paleopressure of <2.1 bar and a probable limit of <0.52 to 1.2 bar (*57*). [Criticism of the raindrop paleopressure constraint (*137*) is refuted in (*138*).] Second, the N_2_/^36^Ar ratio in fluid inclusions indicates *p*N_2_ <1.0 bar [2σ] at 3.3 Ga ago and <1.1 bar at 3.5 to 3.0 Ga ago (*48*, *49*). Third, vesicle volumes in 2.7-Ga-old basaltic lava flows erupted at sea level imply a 0.23 ± 0.23–bar [2σ] paleopressure (*56*).

The inferred history of pN_2_ depends on how the geologic nitrogen cycle has changed over time. Nitrogen can only greatly accumulate as atmospheric N_2_ or in rocks as ammonium, amide, nitride, or organic nitrogen. Under typical mantle temperatures and redox conditions, volcanic gases contain N_2_, not ammonia [e.g., (*139*), p. 49], and because N_2_ is unreactive, it enters the air. Today, N_2_ is also produced when oxidative weathering of organic matter on the continents makes nitrate (NO_3_^−^) that undergoes rapid biological denitrification into N_2_ (*140*). Within large uncertainties, volcanic and oxidative weathering inputs of N_2_ are comparable [(*64*), p. 204]. Using sedimentary C/N data, Berner (*140*) argues that the sum of these N_2_ sources was balanced over the Phanerozoic primarily by N burial in organic matter, so that the Phanerozoic partial pressure of nitrogen, *p*N_2_, varied little.

Som *et al.* (*56*) proposed that low Archean paleopressure arose because today’s long-term N_2_ atmospheric input from oxidative weathering and denitrification was absent. If so, *p*N_2_ would have risen at the GOE, and nitrogen in today’s air must have been in solid phases previously. Certainly on modern Earth, nitrification, by humankind’s addition of nitrate to land and sea, has enhanced denitrification, indicated by increased atmospheric nitrous oxide (N_2_O) [e.g., (*141*), chap. 6]. On the other hand, a model can be constructed where *p*N_2_ declines after the GOE if burial of organic nitrogen increased (*142*).

In the Hadean, *p*N_2_ either started high and then diminished (*143*) or was initially low if nitrogen partitioned into a very reducing magma ocean (*39*). However, low N/C in today’s mid-ocean ridge source basalts (*144*) suggests considerable N_2_ degassing once the upper mantle became oxidized because then nitrogen became insoluble in magmas and upper mantle fluids (*145*). Even today, some of the upper mantle lies within the stability field for ammonium, so that increased oxidation of the early mantle and mantle wedge could have caused more subducted nitrogen to outgas as N_2_ (*145*).

Marine phyllosilicates at 3.8 Ga ago are ammonium enriched (*146*, *147*), which probably came from porewater ammonium (${\text{NH}}_{4}^{+}$) derived from degraded organics (Table 1) (*148*, *149*), and these data have been used to argue that Archean N_2_ was sequestered into solid phases after an early advent of biological nitrogen fixation (*56*, *150*). In the early ocean, ${\text{NH}}_{4}^{+}$ would have been the stable form of dissolved nitrogen unlike today’s nitrate. Consequently, a postulated drawdown of Archean N_2_ involves biological fixation, organic burial, and subduction of ammonium in refractory minerals. The rate of organic burial must have been relatively high for a time-integrated loss to affect *p*N_2_ significantly (*150*), which is not necessarily inconsistent with carbon isotopic constraints because early high degassing of carbon required more carbon to be buried (*130*).

Nitrogen isotopes appear to confirm that biologically fixed nitrogen entered the Archean mantle. Sedimentary organics have δ^15^N = 7 ± 1‰ compared to 0‰ in N_2_ in modern and ancient air and − 5 ± 2‰ in the mantle (*151*, *152*). Fractionation mostly arises when denitrification preferentially converts nitrate or nitrite ^14^N into N_2_. Thus, heavy δ^15^N in 3.1 to 3.5 Ga-old mantle-derived diamonds may be a sedimentary component (*153*).

Ammonium substitutes for potassium, and breakdown of previously subducted ammonium-containing minerals in magmas at oceanic islands releases N_2_ and radiogenic ^40^Ar derived from ^40^K. The ratio ^40^Ar/N_2_ in plume-related lavas scatters by a factor of ~4 to 5, and higher values (older from more ^40^Ar) correlate with smaller, Archean-like values of δ^15^N, consistent with a history of ammonium subduction. Because N_2_ is uncorrelated with nonradiogenic ^38^Ar or ^36^Ar, nitrogen in the current mantle is not primordial but recycled (*154*).

Some use N ingassing versus outgassing fluxes to infer past *p*N_2_. Mallik *et al.* (*155*) estimate modern subduction of 6.4 ± 1.4 × 10^10^ mol N year^−1^ and, with a global degassing flux of 2 × 10^10^ mol N year^−1^ from (*156*), argue that net N ingassing today means higher past pN_2_. However, other outgassing estimates are 7 × 10^10^ mol N year^−1^ from arcs (*157*) or 9 ± 4 × 10^10^ mol N year^−1^ globally [(*64*), p. 204], such that current source and sink N fluxes balance within uncertainties.

Unlike N_2_, other nitrogen-bearing Archean gases would have been trace quantities. With only tiny atmospheric fluxes of nitrate or nitrite, N_2_O from denitrification would have been negligible, perhaps restricted to lakes (*158*). Lightning production of NO, followed by H addition and HNO dissolution and decomposition, might maintain ground-level N_2_O to a few parts per billion by volume (ppbv), compared to a preindustrial ~270-ppbv N_2_O (*159*). The other nitrogen oxides (NO and NO_2_) would have been at trace levels because their lightning production in CO_2_-N_2_ air is inefficient (*160*).

Ammonia (NH_3_) levels of 10 to 100 ppmv would provide a greenhouse effect to counteract the faint young Sun (FYS) (*14*), but these levels of NH_3_ cannot be sustained against UV photolysis (*161*). Possibly, a stratospheric organic haze, such as that on Saturn’s moon, Titan, shielded tropospheric NH_3_ from UV (*162*). However, whether this shielding actually occurred depends on whether hazes actually existed and on the size and radiative properties of haze particles, which remain uncertain (*1*, *163*).

Another nitrogen-bearing gas, hydrogen cyanide (HCN) is more stable photochemically than NH_3_ and made in reducing atmospheres by lightning, impacts (*164*, *165*), or UV-driven photochemistry. In particular, N atoms from N_2_ photolysis in the upper atmosphere can mix to lower levels and react with CH_4_ photolysis products to make HCN (*166*, *167*). At Archean biogenic methane levels of ~10^3^ ppmv, HCN concentrations reach ~10^2^ ppmv.

### Carbon gases in the Archean: CO_2_, CH_4_, and CO

Let us now consider carbon gases, starting with carbon dioxide. Since the early Hadean, CO_2_ has probably always been Earth’s most important noncondensable greenhouse gas. CO_2_ also affects seawater pH and influences the carbon cycle through the formation of carbonates and organic matter. However, direct evidence for Archean CO_2_ levels remains scanty.

**Fig. 3:**
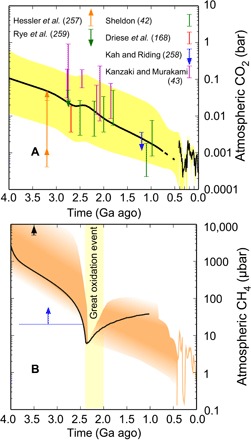
History of CO_2_ and a CH_4_ schematic since the Archean. (**A**) The black line is median CO_2_ from a carbonate-silicate climate model, and yellow shading indicates its 95% confidence interval (*34*); this curve merges with a fit to CO_2_ proxy estimates for 0.42 Ga ago to present from (*256*). Various Precambrian *p*CO_2_ proxy estimates are shown (*42*, *43*, *168*, *257*–*259*). (**B**) A very schematic history of CH_4_. Constraints include a lower limit (blue) required for Archean S-MIF (*44*) and a tentative lower limit of ~3.5 Ga ago from a preliminary interpretation of xenon isotopes (black) (*187*). The black curve is from a biogeochemical box model coupled to photochemistry (*121*). Orange shading is schematic but consistent with possible biological CH_4_ fluxes into atmospheres of rising O_2_ levels at the GOE and in the Neoproterozoic. Note that the suggestion that moderately high levels of methane may have contributed to greenhouse warming in the Proterozoic (*260*, *261*) has been disputed (*262*, *263*) and may depend on fluxes from sources on land (*264*). The curve for ~0.4 Ga ago to present is from (*265*).

Paleosols provide some estimates. Acid leaching in Archean soils arose from CO_2_ dissolved in rainwater. Mass-balance calculations give 10 to 50 PAL of CO_2_ at 2.7 Ga ago and $23_{÷3}^{\times 3}$ PAL of CO_2_ at 2.2 Ga ago (*42*, *168*). However, these analyses assume that all the CO_2_ that entered the soils caused dissolution, so *p*CO_2_ could have been higher if only a fraction of CO_2_ had been used. Another study used an analysis with an estimate of the composition of temperature-dependent aqueous solutions during weathering and, because of a weaker dependence of weathering on *p*CO_2_, obtained higher *p*CO_2_ of 85 to 510 PAL at 2.77 Ga ago, 78 to 2500 PAL at 2.75 Ga ago, and 160 to 490 PAL at 2.46 Ga ago (Fig. 3A) (*43*).

High Archean *p*CO_2_ does not have to induce acidic seawater and dissolve marine carbonates. Instead, an increase in Ca^2+^ concentrations could maintain an ocean saturated in calcium carbonate at alkaline pH; alternatively, seawater pH could be slightly lower than today, but calcium carbonate would remain saturated. In fact, sedimentary marine carbonates appear from 3.52 Ga ago onward [(*98*) and references therein].

Calcium isotopes might provide insight into coupled Archean seawater pH and *p*CO_2_. These two variables and carbonate alkalinity (*Alk* = [${\text{HCO}}_{3}^{-}$] + 2[${\text{CO}}_{3}^{-}$]) define a system where any two variables imply the third. In evaporating seawater, Ca isotopes could undergo Rayleigh distillation if [Ca^2^+^^] << *Alk*, but limestones from 2.6-Ga-old Campbellrand marine evaporites show no spread in δ^44/40^Ca, which might imply [Ca^2^+^^] >> *Alk* and pH of 6.4 to 7.4 for a likely range of Neoarchean *p*CO_2_ (Table 1) (*169*).

Siderite (FeCO_3_) in Archean IFs has been proposed as a *p*CO_2_ proxy if it precipitated in equilibrium with the atmosphere (*170*, *171*). However, data suggest that this siderite was diagenetic (*172*, *173*), so we omit this proxy from Fig. 3A and Table 1.

Potentially, two negative feedbacks control long-term *p*CO_2_. First, the net consumption of CO_2_ in acid weathering of continents or the seafloor ends up making carbonates (*174*)

$$X{\text{SiO}}_{3}+{\text{CO}}_{2}+H_{2}O⇌X{\text{CO}}_{3}+{\text{SiO}}_{2}+H_{2}O$$ (2)

where *X* is a cation. Seafloor weathering occurs when water in the permeable abyssal plains dissolves basaltic minerals, releasing calcium ions and precipitating calcium carbonate in veins and pores (*175*). Second, “reverse weathering” (RW) reactions have been proposed (*176*). Schematically, in net, we represent continental (or seafloor) weathering plus RW, as follows

$$X{\text{SiO}}_{3}+{\text{CO}}_{2}+H_{2}O+\text{Al}{\left(\text{OH}\right)}_{3}⇌X{\text{AlSiO}}_{2}{\left(\text{OH}\right)}_{5}+{\text{CO}}_{2}$$ (3)

This reaction consumes aqueous SiO_2_ and uses cations to make aluminosilicate clays instead of carbonates that consume carbon, so that CO_2_ stays in the atmosphere. High dissolved seawater silica and pH are hypothesized to promote RW and create clay minerals.

If atmospheric *p*CO_2_ is high, pH is low, and temperature is warm, reaction 2 is favored, and *p*CO_2_ falls, in negative feedback on *p*CO_2_ and climate. However, if *p*CO_2_ is low and pH is high, RW, reaction 3, may be an alternative to reaction 2 and a negative feedback on low *p*CO_2_.

Estimates of today’s RW flux (mol Si/year) vary an order of magnitude (*177*, *178*), the rate coefficient for RW reactions varies by many orders of magnitude (*179*, *180*), and the solubility of authigenic phyllosilicates (needed to calculate RW) varies by over an order of magnitude. Consequently, a self-consistent, coupled carbon-silica cycle model since 4 Ga ago shows that RW can be important or unimportant for the Proterozoic climate depending on parameter choice, while RW in the Archean is muted because of probably lower land fraction and sedimentation rate (*181*). Considering these factors, estimates of Archean *p*CO_2_ and seawater pH that we give henceforth are based on carbonate-silicate cycle models without RW.

In the Archean, with greater seafloor production than today, seafloor weathering could have been comparable to continental weathering (*34*, *35*, *182*), and negative feedback (Eq. 2) likely maintained average Archean surface temperatures between 0° and 40°C with seawater pH 6.4 to 7.4 (*34*). The corresponding *p*CO_2_ would have been 0.006 to 0.6 bar 4 Ga ago, assuming that ~10^4^-ppmv CH_4_ also contributed to the greenhouse effect.

Anoxic Archean air could hold 1000 s of parts per million by volume of CH_4_ if a microbial flux of CH_4_ was comparable to today’s (*135*, *183*, *184*). Phylogenetically, methanogens date back to >3.5 Ga ago (*23*). In contrast, the modern oxygenated atmosphere destroys reducing gases rapidly, limiting tropospheric CH_4_ and H_2_ abundances to 1.8 and 0.55 ppmv, respectively.

Evidence points to high levels of Archean CH_4_ (Fig. 3B). First, signs of methanogens and methanotrophs from light carbon isotopes in Archean organics imply methane’s presence [e.g., (*109*)]. Second, Archean S-MIF requires >20-ppmv CH_4_ to generate particulate sulfur, S_8_ (Table 1). Third, the deuterium-to-hydrogen (D/H) ratio of 3.7 Ga-old seawater estimated from serpentine minerals is 2.5% lighter than today, which could be explained by rapid Archean escape of hydrogen and isotopic fractionation (*185*, *186*). This hydrogen was likely derived from UV photolysis of CH_4_ in the upper atmosphere (*135*). Later, we discuss how fractionation of xenon isotopes in the Archean suggests that, ~3.5-Ga ago, CH_4_ levels were >0.5%, i.e., >5000 ppmv (*187*).

Fourth, globally extensive glaciations during the GOE [e.g., (*188*)] provide circumstantial evidence for high Archean CH_4_. At the tipping point, air flips from anoxic to oxic in only ~10^4^ years, causing a ~10°C temperature drop by oxidizing CH_4_. This chemical transition is far faster than the ~10^5^- to 10^6^-year response of the carbonate-silicate thermostat.

Another carbon-containing gas, carbon monoxide (CO), was probably not abundant in the presence of an Archean microbial biosphere. The Last Universal Common Ancestor was likely capable of anaerobic CO consumption (*189*), which involves water as a substrate and catalysts such as iron sulfide that were probably widespread

$$H_{2}O+\text{CO}\to {\text{CO}}_{2}+H_{2}$$ (4)

With this reaction, microbes would draw CO down to 10^0^ to 10^2^ ppmv (*183*). However, episodic CO levels at percent levels may have occurred when large impacts delivered cometary CO ice or organic matter that was oxidized (*190*).

### A high-altitude Archean organic haze?

Because of relatively abundant CH_4_, a high-altitude Archean organic haze might have formed, as mentioned earlier. The idea was first suggested in the 1980s (*191*). Empirically, if the CH_4_:CO_2_ ratio exceeds ~0.1 in a UV-irradiated CO_2_-N_2_-CH_4_ mixture, radicals from methane photolysis polymerize into organic particles (*192*).

When and whether an organic haze formed are uncertain. A haze could have affected tropospheric sulfur gases by blocking UV photons. Consequently, the structure of S-MIF variations in Archean sedimentary minerals and their correlation with light, organic δ^13^C have been attributed to episodic hazes driven by variable atmospheric CH_4_:CO_2_ ratios (*60*, *193*–*196*). However, given—dare we say—only a hazy understanding of which species and reactions are important for S-MIF (see earlier), interpretations of episodic hazes are permissive rather than definitive.

### Hydrogen abundance

The Archean lower atmosphere is unlikely to have been H_2_-rich given the antiquity of methanogens (*23*, *51*, *197*), some of which convert H_2_ into methane through a net reaction

$$4H_{2}+{\text{CO}}_{2}\to {\text{CH}}_{4}+2H_{2}O$$ (5)

Anoxygenic photosynthesis also consumes H_2_ [e.g., (*198*)]. In models with methanogens and H_2_-based photosynthesizers, atmospheric H_2_ mixing ratios depend on assumed H_2_ outgassing and biological productivity but generally are ≤10^−4^ (*183*, *184*). These levels preclude H_2_ as an important Archean greenhouse gas. Detrital magnetite carried in rivers 3.0- to 2.7-Ga ago would have dissolved at high pH_2_ via microbial reduction of Fe^3^+^^ to soluble Fe^2^+^^ using H_2_ (*199*), providing an upper limit of pH_2_ ≤ 10^−2^ bar (*200*).

### Xenon isotopic constraints on oxygen, hydrogen, and methane levels, plus Earth’s oxidation

Changes in atmospheric Xe isotopes through the Archean stop after the GOE (analogous to S-MIF) (*49*) and potentially tell us about O_2_, CH_4_, and H_2_ levels (*187*), including in the otherwise hidden Hadean, as discussed below. The trend also relates to how much hydrogen escaped from Earth and hence Earth’s total oxidation over time.

**Fig. 4:**
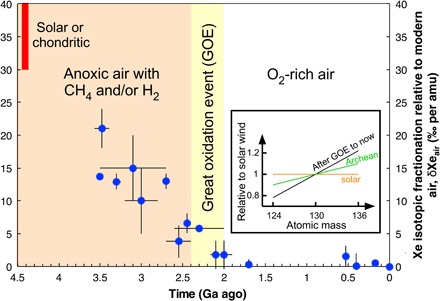
Mass fractionation of nine atmospheric xenon isotopes over time relative to modern air per atomic mass unit showing relative enrichment in light isotopes in the past. Data from (*49*). The vertical axis shows the fractionation per atomic mass unit (amu) of atmospheric xenon relative to modern air. To compute this average fractionation across the nine isotopes, Avice and co-workers (*49*) normalized the isotopic compositions to ^130^Xe and to the isotopic composition of the modern atmosphere using the delta notation. For a Xe isotope of mass *i*, δ*^i^*Xe_air_ = 1000 × ((*^i^*Xe/^130^Xe)_sample_/(*^i^*Xe/^130^Xe)_air_ − 1). The slope of a straight line fit to the normalized data provides the average fractionation per atomic mass per unit and its uncertainty, i.e., plotted points. Inset: A diagram showing schematically how the slope of the fractionation of the nine isotopes changed over time relative to the initial solar composition, where the graph is normalized to atomic mass 130.

Xenon in fluid inclusions in Archean rocks becomes isotopically heavier through the Archean relative to an initial solar composition until the fractionation reaches that of modern air around 2.1 to 2.0 Ga ago (Fig. 4) (*49*). Xenon dragged out into space by escaping hydrogen during the Archean and Hadean best explains the progressive mass fractionation (*49*, *187*). An alternative explanation from trapping of Xe^+^ in organic hazes (*201*) has the problem that weathering or microbial processing of buried organics would release xenon and modulate the Xe isotopes in post-Archean air, which is not observed (*202*).

Today, the nine atmospheric Xe isotopes [124 to 136 atomic mass units (amu)], have a huge fractionation of 4.2% per amu relative to solar or chondritic sources, whereas the six, lighter krypton isotopes (76 to 86 amu) are barely fractionated. Earth’s Xr/Kr ratio is also 4 to 20 times less than meteorites, implying selective Xe loss.

Unlike lighter krypton, xenon is easily ionized by solar UV or charge exchange with H^+^ ions, so Xe^+^ can be dragged out to space by escaping H^+^ ions (*187*). Whereas Xe^+^ is unreactive with H, H_2_, or CO_2_ (*203*), any Kr^+^ ions are neutralized via Kr^+^ + H_2_→KrH^+^ + H and KrH^+^ + *e*^−^→Kr + H, explaining the lack of Kr isotope fractionation. Ions are tethered to Earth’s magnetic field lines, but a “polar wind” of hydrogen ions escapes along open field lines at the poles, accounting for ~15% of all hydrogen escapes today. Xe^+^ ions could be dragged by a vigorous ancient polar wind. That requires copious hydrogen to be derived from relatively abundant CH_4_ and/or H_2_ in the lower atmosphere. UV decomposes CH_4_ in an anoxic upper atmosphere, releasing hydrogen [e.g., (*166*)].

The hypothesized xenon escape works only in anoxic air, so, like S-MIF, Xe isotopes record the GOE (Fig. 4). Oxic air destroys H_2_ and CH_4_, making their abundances too low to supply enough hydrogen to drag along xenon. In addition, O_2_ would remove Xe ions in a resonant charge exchange reaction (*203*)

$${\text{Xe}}^{+}+O_{2}\to \text{Xe}+O_{2}^{+}$$ (6)

Our preliminary model finds that Xe can escape when the total hydrogen mixing ratio exceeds ~1% for the solar extreme UV flux expected around ~3.5 Ga ago (*187*). The total hydrogen mixing ratio, *f*_T_(H_2_), in equivalent H_2_ molecules, is

$$f_{T}(H_{2})=0.5f_{H}+f_{H_{2}}+f_{H_{2}O}+2f_{{\text{CH}}_{4}}+\ldots$$ (7)

Thus, if nearly all Archean hydrogen was biologically converted into CH_4_ (Eq. 5), we interpret the xenon escape constraint of >1% *f*_T_(H_2_) to be >0.5% CH_4_, which is a rare empirical constraint on Archean CH_4_ (Fig. 3B).

The inferred escape of a strong reductant, hydrogen, would oxidize entire Earth. The total oxidation during the Archean is equivalent to oxygen from a tenth of more of an ocean (*187*).

### Sulfur gases

S-MIF proves that Archean S-containing gases existed but tells us little about their concentrations. All sulfur gases apart from S_8_, which condenses, are susceptible to UV photolysis and are short lived and interconverting. Consequently, atmospheric sulfur is sequestered into stable sulfuric acid aerosols by oxidation or S_8_ aerosols by reduction. For a wide range of Archean atmospheric redox conditions, both types of aerosol should form and precipitate (*44*).

Although sulfur gases absorb UV, they probably did not shield Earth’s surface. Surface temperatures greater than ~50°C would be required to produce enough atmospheric S_8_ vapor to shield surface life from UV (*204*).

## THE ARCHEAN CLIMATE

### The Faint Young Sun problem

Related to atmospheric composition, discussed above, is another basic problem: How the Archean climate remained clement under a fainter Sun. In the Sun’s core, nuclear reactions fuse four protons into helium nuclei, increasing the mean molecular mass and decreasing the number of particles per unit volume. In response, the weight of the overlying column presses inward, and the core temperature rises. A greater radial temperature gradient drives more outward radiation flux, so the Sun brightens. Solar evolution models show that the Sun was 25 to 30% fainter at 4 Ga ago (*12*, *13*, *15*).

One solution to the FYS is that the young Sun was not faint but more massive and therefore as bright as today (*205*, *206*). The Sun would then need to lose enough mass over time so that a declining weight of the column above the core matched the pressure loss from particles fused in the core. However, observations are unsupportive: Fast mass loss only happens in the first ~200 Ma after Sun-like stars form (*207*, *208*).

As well as solar luminosity, Earth’s mean global temperature depends on the Bond albedo (the reflectivity over all wavelengths) and greenhouse effect [e.g., (*64*), pp. 34 to 36]. Some FYS hypotheses invoke clouds: that the Archean Bond albedo was low because of substantially different cloud cover (*170*, *209*) or that abundant high-altitude cirrus clouds warmed early Earth (*210*). However, no plausible combination of few low clouds (which tend to reflect sunlight) and many high, icy clouds (which tend to warm the surface with downwelling infrared) solves the FYS problem (*211*). Three-dimensional climate models with a lower solar flux produce weaker evaporation and fewer low clouds, but a strong greenhouse effect is still required because the albedo decrease is small (*212*, *213*).

The most plausible solution to the FYS is a big greenhouse effect (*14*, *214*, *215*). Water vapor is an important greenhouse gas but, on its own, cannot solve the FYS problem. Water vapor condenses into rain and snow, so its abundance is limited by the saturation vapor pressure that depends only on temperature, which is set by noncondensable greenhouse gases, such as CO_2_ and CH_4_.

Consequently, substantial CO_2_ is the most obvious driver of an enhanced Archean greenhouse effect. We would expect Archean *p*CO_2_ to have been high because of Earth’s carbonate-silicate cycle thermostat (Eq. 2) (*174*). If global temperatures and CO_2_ are low, CO_2_ removal via rainfall is slow, as are rates of continental and seafloor silicate weathering. Then, geological emissions of CO_2_ raise atmospheric CO_2_ levels, increasing global temperatures. Conversely, if the climate warms too much, greater rainfall and silicate weathering consume CO_2_ and cool Earth.

These negative feedbacks would have moderated the Archean climate to a mean global temperature of 0° to 40°C (or 0° to 50°C without land and only seafloor weathering) (*34*). CO_2_ alone could have solved the FYS problem with 0.004- to 0.03-bar CO_2_ at 2.5 Ga ago and 0.024- to 1-bar CO_2_ at 4 Ga ago, where the spread comes from uncertain carbonate-silicate model parameters.

However, without O_2_ in the atmosphere, a CH_4_ greenhouse bears consideration. Methane levels of 10^3^ to 10^4^ ppmv produce ~10 to 15 K of Archean greenhouse warming, which lowers the *p*CO_2_ needed to warm the Archean Earth (Table 1). Some of this warming comes from a few parts per million by volume of ethane, C_2_H_6_, which is derived from CH_4_ (*215*).

Both substantial CH_4_ and CO_2_ may be necessary if lower bounds on *p*N_2_ discussed earlier are valid. Although N_2_ itself is not an effective greenhouse gas, it pressure broadens line and continuum infrared absorption, enhancing the greenhouse effect. If Archean *p*N_2_ was about half of today, a few kelvin would be lost from the greenhouse effect (*143*).

However, too much CH_4_ relative to CO_2_ would create the high-altitude organic haze mentioned earlier, which may cool Earth by up to ~20 K (*1*, *215*) by absorbing incoming solar radiation and radiating energy back to space in a so-called “anti-greenhouse effect” (*216*). A cooling limit exists because as a haze becomes more UV absorbing, it shields CH_4_ molecules from the photolysis needed for further haze formation. Thus, attenuation of sunlight saturates (*1*, *163*).

Earlier, we noted that an organic haze might protect tropospheric ammonia from UV (*162*), but whether NH_3_ was a viable greenhouse gas is unclear. The composition of organic aerosols in the Archean where sulfur was present and the C/O ratio was <1 was different from particles on Titan where sulfur is absent and C/O > 1. Consequently, the UV attenuation properties of early Earth haze particles remain uncertain.

Another proposed early greenhouse gas is H_2_, if it attained percentage abundances (*14*, *217*, *218*). Molecular collisions produce temporary charge separation, allowing H_2_ to undergo collision-induced absorption of infrared photons. For reasons given previously, Archean H_2_ is unlikely to have been abundant. Instead, H_2_ may be relevant for the Hadean greenhouse, if percentage H_2_ levels arose from H_2_ outgassing or impacts.

Sulfur gases were likely unimportant for warming. SO_2_ is a strong greenhouse gas, but tropospheric SO_2_ dissolves in rainwater, and Archean stratospheric SO_2_ photochemically disproportionates into UV-stable polysulfur and sulfuric acid aerosols, as noted previously. The latter raises the albedo and more than offsets any SO_2_ greenhouse warming. H_2_S is a weak greenhouse gas that overlaps in the absorption with CH_4_ and is UV fragile. Carbonyl sulfide (OCS) has been suggested as an Archean greenhouse gas because UV photolysis of OCS was posited to explain observed relationships in S-MIF (*219*). OCS persists today because it is not oxidized by OH radicals that cleanse the troposphere of other pollutants. However, in the absence of an ozone shield, OCS is susceptible to photolysis and cannot build up significantly (*46*).

Last, an alternative to the carbonate-silicate cycle stabilization of Earth’s climate is the “Gaia hypothesis,” which proposes that life interacts with inorganic Earth as a self-stabilizing system that maintains habitable conditions [e.g., (*220*)]. The Archean biosphere was surely important for atmospheric composition, which intertwines Earth’s inhabitance and habitability. However, debate continues about if and how adaptive evolution of the biosphere can produce a more stable climate than an abiotic Earth (*221*, *222*).

### Physical and geochemical evidence of a moderate Archean climate

Let us now consider proxy data for the Archean climate. Glacial rocks are one line of evidence. These include dropstones in sediments from melted icebergs, unsorted clasts mixed with silt and clay deposited at the base of glaciers that are preserved as diamictites, and striations made by rocks embedded in moving glaciers that scoured underlying surfaces. In the Archean, any indication of polar environments from glacial rocks would suggest a relatively cool world given that since ~34 Ma, a climate with poles covered in ice has required average global temperatures below ~20°C (*223*).

Glacial rocks are reported from 3.5, 2.9, and 2.7 Ga ago. The oldest, from the Barberton of South Africa, includes clasts in finely laminated sediments, interpreted as dropstones, found below diamictites (*55*). Later, in the ~2.9-Ga-old Pongola in South Africa, which was 43° to 48° paleolatitude (*224*), diamictites contain striated clasts, while associated silty laminates have dropstones with splash-up of substrata (*54*). Then, at 2.7 Ga ago, diamictites with dropstones occur in India (*225*) and Montana (*226*).

Another line of evidence about paleotemperature are ratios of ^18^O/^16^O in marine cherts and carbonates. These ratios decline with increasing age, which, at face value, suggests Archean ocean temperatures of 50° to 85°C (*227*). Cherts and carbonates precipitated from seawater acquire less ^18^O relative to the seawater as temperature increases because, at equilibrium, warmth allows stronger ^18^O bonds to be broken and replaced with ^16^O. Not all Archean isotopic studies infer hot temperatures, however. Combined O and H isotope ratios have suggested surface temperatures <40°C (*52*), while O isotopes in Archean phosphates have produced 26° to 35°C upper limits (*53*).

Most researchers have doubted the isotopic inferences of persistently high Archean surface temperatures because the climate was sometimes cold enough for the aforementioned glaciations, and chemical weathering of quartz from hot climates is lacking (*228*). Two alternative interpretations are as follows: (i) Archean surface temperatures were similar to today’s because the oxygen isotope composition of seawater, δ^18^O_seawater_, increased by ~15‰ since 3.5 Ga ago (*229*, *230*). (ii) Older cherts and carbonates have nonprimary δ^18^O from high alteration temperatures or hydrothermal fluids (*55*, *231*).

The first explanation relies on changes in the relative rates of high and low temperature water-rock interactions. Hydrothermal seafloor interactions increase δ^18^O_seawater_, whereas low-temperature seafloor and continental weathering decrease δ^18^O_seawater_. Archean oceans with shallow seafloor hydrothermal circulation and an increase in Phanerozoic pelagic sediments that lowers seafloor weathering might cause secular increase in δ^18^O_seawater_ [e.g., (*229*)]. However, measurements find ancient δ^18^O_seawater_ ~0‰ (Vienna standard mean ocean water) in 2-Ga-old ophiolites (*232*), 3.8-Ga-old serpentine minerals (*185*), Archean pillow basalts (*233*), and kerogens in Archean cherts (*234*). In addition, clumped isotopes show little change in Phanerozoic δ^18^O_seawater_ [e.g., (*235*)]. Alternatively, O isotopes in iron oxides since 2 Ga ago suggest a secular increase in δ^18^O_seawater_ (*236*).

The second possibility is that δ^18^O of cherts and carbonates is more altered with age. Microanalysis shows how chert replaced sedimentary carbonates and did not precipitate from seawater (*231*). In addition, stringent geochemical and petrographic selection criteria for paleothermometry eliminate Archean cherts (*237*). The same issue applies for silicon isotopes in Archean cherts, which have been used to infer hot surface temperatures (*238*). Last, the triple O isotope composition of Archean cherts cannot be reconciled with equilibrium precipitation from seawater and requires alteration (*239*).

Phylogenetic inferences of early thermophilic microbes have also been used to argue for hot Archean oceans (*240*, *241*). However, other more convincing reconstructions and biochemical considerations suggest a mesophilic (<50°C) common ancestor (*242*, *243*).

Temperature limits on mineral formation may also favor a temperate Paleoarchean. Pseudomorphs of barite after gypsum are reported in 3.5-Ga-old evaporites that are now partially silicified (*244*). In halite-saturated brines, gypsum (CaSO_4_.2H_2_O) forms at <18°C; otherwise, anhydrite (CaSO_4_) precipitates (*245*). It has been claimed from x-ray tomography that hydrothermal barite, not gypsum, was actually primary (*246*). However, three-dimensional universal stage petrography found interfacial angles diagnostic of gypsum at the original crystal faces (*244*), which are partially replaced by quartz and draped by quartz, so cannot show up in x-ray density contrasts.

### Additional external parameters and their effect on Archean climate

Additional factors may have affected the Archean climate. One external parameter was a faster rotation of early Earth. Today, the Moon recedes ~4 cm year^−1^ from Earth due to tides, so Earth is despinning over time to conserve angular momentum in the Earth-Moon system. Going back in time, depositional cycles in the 2.45-Ga-old banded iron Weeli Wolli Formation, Australia indicate a ~17-hour day (*247*).

In general, a faster Archean rotation rate reduces heat transport from equator to pole (*212*, *213*), particularly if the atmosphere had less mass (*248*). Thus, warm tropics and glaciated poles would arise if the Archean had a thin atmosphere.

External parameters that modulate the carbonate-silicate cycle would also affect the long-term climate. These include land fraction, CO_2_ outgassing, and biological modification of weathering. In general, how CO_2_ outgassing changed with time dominates the uncertainty (*34*).

## CONCLUSIONS AND OUTLOOK

**Fig. 5:**
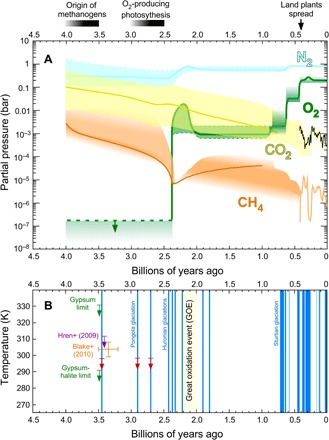
An overview of post-Archean atmospheric evolution in the context of biological evolution and constraints on mean global temperature in the Archean (see text) in the context of the glacial record. (**A**) Uncertainties on gas concentrations are a factor of a few or more as detailed in Table 1, the text, and the other figures. Dinitrogen may have tracked O_2_ levels due to an oxidative weathering and denitrification source of N_2_, but *p*N_2_ changes are debated. Methane was oxidized as O_2_ rose but could have been protected subsequently under an ozone layer, depending on post-Archean CH_4_ source fluxes. The secular decline of CO_2_ is a feedback effect in the geological carbon cycle induced by decreasing solar luminosity. (**B**) Constraints on Archean mean global temperature. Vertical blue bars denote that glacial rocks exist, noting that the durations of glaciations in the early Proterozoic and earlier are poorly known. Neoproterozoic and Phanerozoic glaciation ages are from (*266*, *267*). A proposed Mesoproterozoic glaciation (*268*) is not plotted because its age is disputed and possibly Sturtian (*269*). Cenozoic glaciations only occur at a global mean temperature below ~20°C. Red arrows on the Archean glaciations are a more conservative 25°C upper limit, taking into account of the possible effects of different land configurations and lack of vegetation. Low CO_2_ during the Phanerozoic (A) correlates with glaciations (B), such as Carboniferous-Permian ones, 335 to 256 Ma ago. Precambrian greenhouse gases must also have fluctuated, but the amount is unknown and so not reflected in (A).

The general view that emerges is an Archean atmosphere devoid of O_2_ and enriched in CO_2_ and CH_4_ greenhouse gases that countered the FYS. N_2_ was a bulk gas, but proxy data, although debated, suggest that N_2_ levels were similar to today or possibly a factor of a few lower, which, if correct, requires understanding of how the nitrogen cycle operated differently in an anoxic world. With the present information, a schematic overview of atmospheric evolution is shown in Fig. 5A.

The Archean climate was probably mostly moderate (Fig. 5B). Although some argue for a hot Archean climate, glacial rocks at 2.7, 2.9, and 3.5 Ga ago and recent isotopic analyses call that idea into question; indeed, our current understanding of feedbacks in the geologic carbon cycle suggests surface temperatures within 0° to 40°C.

Reconstructing the history of O_2_ is informed by proxies, but they are often indirect. Once cyanobacteria evolved, local or regional oxygen oases in lakes or shallow seawater are possible, and various redox-sensitive proxies suggest that these oases actually existed by 3.2 to 2.8 Ga ago under globally anoxic air. The newest relevant data are mass fractionations of xenon isotopes, which gradually increase through the Archean relative to initial solar values. These data are best explained by xenon escaping to space as an ion dragged out by hydrogen originating photolytically from an anoxic atmosphere enriched in methane until the GOE.

Despite improved knowledge of the Archean, a relative dearth of uncontested data that constrain basic environmental variables, such as Archean seawater pH, climatic temperature, barometric pressure, and changing levels of the greenhouse gases CO_2_ and CH_4_ through time, means that more proxy development and measurements are critical. It is also implausible that the Archean—one-third of the history of Earth—had a constant climate. However, our knowledge of Archean climatic variability is meager.

The biosphere was surely a major influence on Archean atmospheric composition, as it is today. Consequently, resolving when key biological innovations evolved—such as nitrogen fixation, methanogenesis, anoxygenic photosynthesis, and oxygenic photosynthesis—and understanding their influence are essential for improving models of atmospheric evolution.

This understanding may help us interpret future exoplanet data because atmospheres on other rocky Earth-sized worlds would initially be anoxic. On Earth, oxygenic photosynthesis evolved only once, perhaps because of its biochemical complexity (*249*). Consequently, if life exists elsewhere, inhabited planets with Archean-like atmospheres may be the most common type. So, determining what the Archean atmosphere was made of, and the influence of life, could help us distinguish biogenic gases in exoplanet atmospheres and so find life elsewhere (*2*, *250*).

Last, the connection between atmospheric evolution and debated trends in solid Earth evolution was outside the scope of this review. However, quantifying temporal changes in land area, surface rock composition, weathering, the size of volcanic and metamorphic fluxes of gases, and the redox evolution of outgassing all need further development to understand their implications for Earth’s atmospheric evolution or to extrapolate to exoplanets.
